# Spatial and trend analysis of gestational syphilis cases in Brazil from 2011 to 2020: an ecological study

**DOI:** 10.1186/s12889-024-19286-z

**Published:** 2024-07-11

**Authors:** Thales Philipe Rodrigues da Silva, Rafaela Siqueira Costa Schreck, Débora Caixeta Bernardes de Oliveira, Letícia Vieira Mascarenhas, Bianca Maria Oliveira Luvisaro, Bárbara Tideman Sartório Camargo, Eunice Francisca Martins, Giselle Lima de Freitas, Fernanda Penido Matozinhos

**Affiliations:** 1 Department of Women’s Health Nursing, Escola Paulista de Enfermagem, Universidade Federal de São Paulo, São Paulo, Brazil; 2 Graduate Nursing Program, School of Nursing, Universidade Federal de Minas Gerais, Belo Horizonte, Brazil; 3 Department of Maternal and Child Nursing and Public Health, School of Nursing, Universidade Federal de Minas Gerais, Avenida Alfredo Balena, 190, Santa Efigênia, Belo Horizonte, MG CEP: 30130-100 Brazil; 4 School of Nursing, Universidade Federal de Minas Gerais, Belo Horizonte, Minas Gerais Brazil; 5 Graduate Nursing Program, Escola Paulista de Enfermagem, Universidade Federal de São Paulo, São Paulo, Brazil

**Keywords:** Syphilis, Gestational syphilis, Pregnant women, Sexually transmitted diseases

## Abstract

**Objectives:**

To analyze the rate of gestational syphilis (GS) based on temporal trends over 11 years, as well as the spatial distribution of GS in Brazil, based on the identification of spatial clusters.

**Methodology:**

An ecological, using Brazil and its regions as an analysis unit, based on gestational syphilis data reported in the Notifiable Diseases Information System (SINAN), from 2011 to 2020. Thematic maps were built for spatial data analysis, and the Prais-Winsten autoregressive model was used to verify the trend. Spatial analysis identified the distribution of clusters (high-high; low-low; high-low and low-high) of distribution of GS across Brazilian municipalities, using a 5% significance level.

**Results:**

Gestational syphilis experienced a considerable increase in cases during the studied period, with a peak of 37,436 cases in 2018. The spatial distribution of the disease is heterogeneous in the country. A growing trend was observed in all states of Brazil, except for Espírito Santo, where it remained stationary, with a monthly variation of 10.32%.

**Conclusion:**

The spatial and temporal trend analysis point to syphilis as an important public health problem. The numbers are alarming and show the urgent need for measures to prevent and control syphilis during pregnancy.

## Background

Sexually transmitted infections (STIs) stand out with a serious global public health problem, with syphilis being the most common worldwide [1], generating economic, social, and health impacts. Every year, syphilis affects more than 12 million people globally [2]. Recent syphilis is defined as the primary, secondary, or latent phases of the disease, periods when syphilis is highly contagious. It can be considered as late syphilis when the disease is in the tertiary or late latent phases. As the syphilis infection progresses from one phase to another without treatment, individuals may develop cardiovascular problems and irreversible neurological complications [3].

Syphilis is caused by the bacterium *Treponema pallidum*, which can be transmitted through sexual intercourse or congenital form during pregnancy, characterized by vertical transmission of the bacterium by pregnant women not treated or treated inappropriately, and is defined as congenital syphilis (CS). Congenital syphilis is the second leading cause of preventable fetal death worldwide, preceded only by malaria [4]. Gestational syphilis (GS), the focus of this study, occurs during infection of the bacteria in the gestational period, delivery or puerperium [3]. It is known that untreated or improperly treated pregnant women have a 50% chance of transmitting the bacteria to their conceptus, and this rate can reach a 70–100% chance if mothers are in the primary phase of the disease. Although the probability is lower and lower, the more advanced the phase of infection and the gestational age are, the higher the chance that the spread of syphilis can occur in any gestational period [5].

In addition to causing prematurity and low birth weight, GS and CS are responsible for a significant portion of fetal losses and neonatal deaths, reaching 40% of children infected with bacteria [6]. These data highlight the need to perform screening of the disease in pregnant women during prenatal care, aiming at the containment of CS [5]. Despite major advances in knowledge and low-cost access to the diagnosis, treatment and cure of the disease, GS remains a public health problem worldwide [7].

In 2016, the World Health Organization (WHO) developed a strategy to combat STIs, prioritizing the elimination of syphilis and aiming at a reduction of 90% in the overall incidence of cases, this is an important milestone of the 2030 Agenda [8]. In contrast, a study on the new cases of CS from 2010 to 2018 indicated that the infection rate by the bacteria remains endemic in low- and middle-income countries, and considerably increasing in high-income countries [8]. The African region, despite having achieved considerable progress, remains particularly affected with a high prevalence of syphilis cases and global representation of this infection. In this global scenario, the main challenges in achieving syphilis control include the implementation of health care actions integrated with surveillance and control, guaranteeing timely diagnosis and treatment [9].

In Brazil the compulsory surveillance of the GS has been carried out since 1986 via the Notifiable Diseases Information System [10, 11]. According to data from 2019, the GS rate was equal to 21.8 cases per 1,000 live births [12]. In 2021, 74 thousand cases of syphilis were registered in pregnant women, with 27 thousand occurrences of CS [13]. The rising trend in these rates show that the disease still presents itself as a challenge throughout the country [14].

This challenge also permeates social inequities, since, in Brazil, syphilis was considered a socially determined disease, associated with social, economic, infrastructure and access to health services factors. In this sense, populations with greater social vulnerability, such as brown and black women and those with low education, are more exposed to difficulties in accessing and receiving qualified health care during prenatal care [15, 16].

The spatial and trend analysis of GS helps to understand the health situation of the Brazilian population, as a useful tool in the prevention and control of GS, helping to adequately control this infection in Brazil. In other words, it is capable of encouraging decision-making on the distribution of resources, according to the areas of greatest vulnerability of the studied indicator. Thus, the importance and potential contribution of this study to achieving the target of reduction of reducing vertical transmission of infection to < 0.5 case for every 1,000 live births [1].

This study aims to analyze the GS rate based on temporal trends over 11 years, as well as the spatial distribution of GS in Brazil, based on the identification of spatial clusters.

## Method

This is an ecological, with a spatial analysis and a trend of cases of gestational syphilis, which were reported in the Notifiable Diseases Information System (SINAN - In Portuguese: *Sistema de Informação de Agravos de Notificação*). It was based on comparative analysis the 26 Federative Units (FU) of Brazil and the Federal District and their respective regions.

SINAN is mainly fed by the notification and investigation of cases of diseases and illnesses on the national list of compulsorily notifiable diseases, contained in Consolidation Ordinance No. 4 of 28 September 2017, but states and municipalities may include other important health problems in their region. Its effective use makes it possible to carry out a dynamic diagnosis of the occurrence of an event in the population, and can provide support for causal explanations of compulsorily notifiable diseases, as well as indicating risks to which people are subject, thus helping to identify the epidemiological reality of a given geographical area. It is therefore an important tool for helping to plan health, prioritize interventions and assess the impact of interventions.

Brazil has a territorial area of 8,510,345.540 km², which makes it the largest country in South America and the fifth largest in the world. The country consists of 26 states, 01 federal district and 5,570 municipalities, and all this territory is divided into five regions: North, Northeast, Center-West, Southeast, and South. It has an estimated population of 213,317,639 people; of these, more than 87% live in urban areas. It is currently the 12th largest economy in the world, with per capita GDP of R$ 35,161.70. As for the HDI, it has an index of 0.765, considered as high human development [17].

This study population consists of all notifications of GS from 2011 to 2020. Data were collected in August 2022, and all variables were collected from the Department of Informatics of the Unified Health System (DATASUS) website [18] (available at: https://indicadoressifilis.aids.gov.br/).

The following data were collected: Municipality of residence, year and SG incidence rates. Data were aggregated as per the country regions (North, Northeast, Center-West, Southeast and South), comprising 26 FUs and the Federal District. As to the descriptive spatial analysis of this study, thematic maps were constructed. The QGIS software (version 3.18.3) was used to create maps.

The data obtained in the study was for the years 2011 to 2020. The method adopted to calculate the incidence rate is described below:

- To calculate the incidence rate of GS in this study, the SINAN calculation method was adopted, with the rate being the product of a division where the numerator was the number of cases reported or confirmed in pregnant women per analysis unit (Brazil, Units Federative and Federal District, regions and municipalities); divided by the denominator the number of live newborns according to their unit of analysis respectively, multiplied by a thousand.

After that, spatial data analysis techniques were used, considering the digital meshes of the municipalities. To verify whether municipalities with similar SG rates formed clusters or spatial clusters, Global Moran’s I was calculated, ranging from − 1 to + 1, with positive values indicating direct autocorrelation and negative values indicating inverse autocorrelation. The spatial autocorrelation is interpreted according to Global Moran’s I, and may be weak (Moran I < 0.3), moderate (Moran I ≥ 0.3; < 0.7) or strong (Moran I ≥ 0.7) [19].

From the cartographic base accessed by the Brazilian municipalities, consulted on the IBGE website, and using Geographic Information Program Systems, cartograms were elaborated to present to the clusters a statistical significance of *p* < 0.05. Using these cartograms (LISA Cluster Map type), low-low (dark blue) spatial clusters were presented, formed by municipalities with a low gestational syphilis incidence rate and surrounded by municipalities with a low gestational syphilis incidence rate; high-high (dark red color), formed by municipalities with a high gestational syphilis incidence rate and surrounded by municipalities that also showed similar behavior; high-low (municipalities with a high incidence rate of gestational syphilis surrounded by municipalities with a low incidence rate of gestational syphilis) and low-high (municipalities with a low incidence rate of gestational syphilis surrounded by municipalities with a high incidence rate of gestational syphilis).

In this study, the Moran’s I significance level of 5% was considered after 9,999 permutations, that is, the areas with statistically significant spatial correlation were those whose *p* value was less than or equal for 0.05 after the 9,999 random permutations for both indexes. The GeoDa software (version 1.20.0.8) was used for this analysis.

To carry out the trend analysis, the Prais-Winsten autoregressive models were used, in which the dependent variables were the incidence rates of GS and, as an independent variable, the years of the study (2011 to 2020).

To perform the Prais-Winsten regression, the GS incidence rates were transformed into the logarithmic scale. This process is carried out to reduce the heterogeneity of the variance of the residues from the time series regression analysis [20].

The calculation of the annual percentage change (APC) was also performed for each dependent variable analyzed. The following formula was used to calculate the APC:

APC = (-1 + 10[B1]*100%), where B1 refers to the angular coefficient (beta) of the Prais-Winsten regression [14].

The 95% confidence intervals (IC95%) of APC measurements were also calculated using the following formula:

IC95% minimum (-1 + 10 ^[B1−t*e]^*100%) and IC95% maximum (-1 + 10 ^[B1+t*e]^*100%).

The values of the angular coefficients (B1) of the Prais-Winsten regression and standard errors were generated by the statistical analysis program. The “t” of the formula refers to the Student’s t-test [20].

The regression results were interpreted as follows: Increasing trend, when the *p*-value was less than 0.05 and the regression coefficient was positive; decreasing trend, when the *p*-value was less than 0.05 and the regression coefficient was negative; or stationary trend, when the *p*-value was greater than 0.05 [20].

Due to the nature of this study of using freely accessible data, available by Datasus, it was not necessary to submit the present study to the Research Ethics Committee, in accordance with Resolution 466/2012 of the National Brazilian Health Council.

## Results

### Spatial distribution of SG in Brazil

**Fig. 1:**
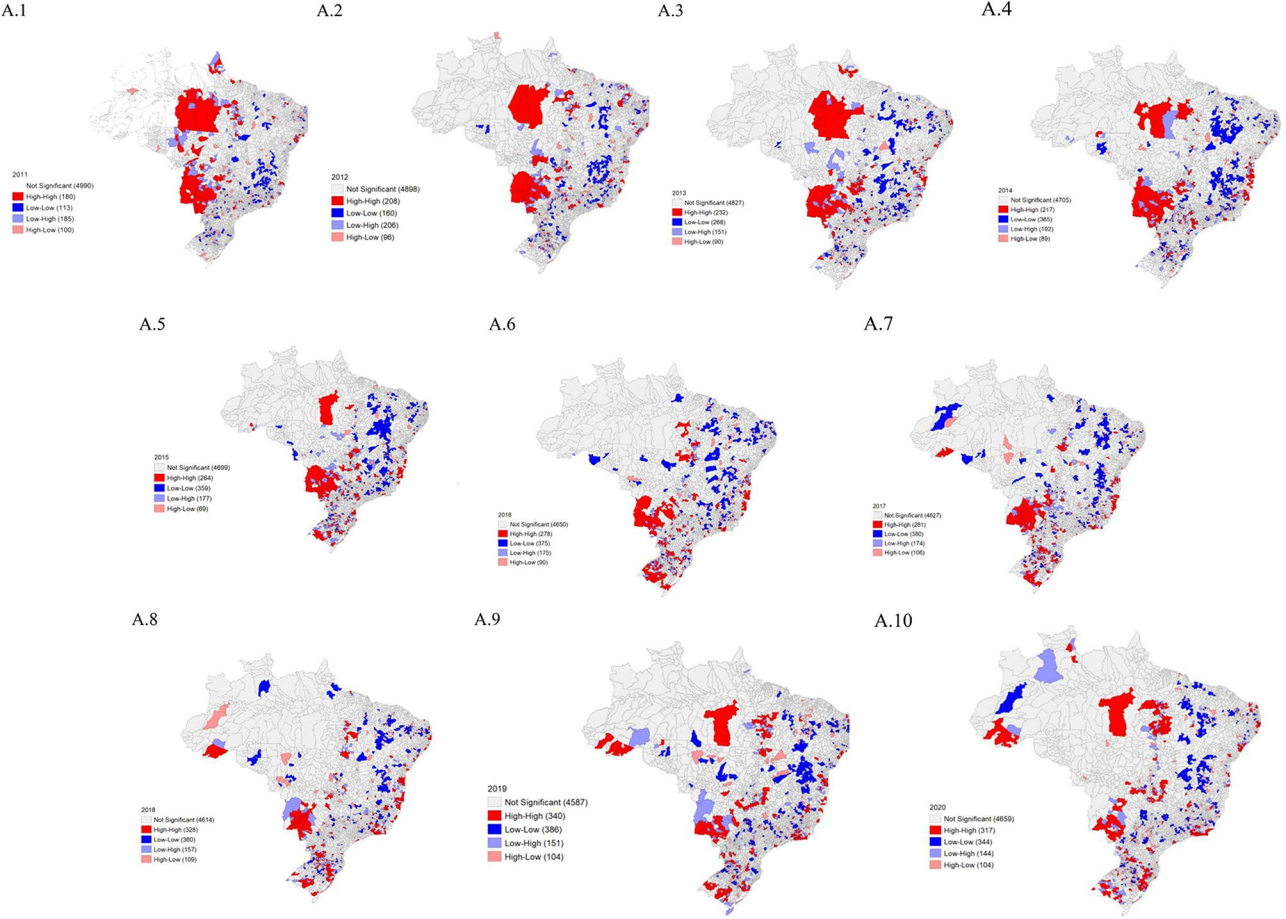
Spatial distribution of gestational syphilis cases in Brazil, from 2011 to 2020

The results of this study showed a progressive increase of high-high clusters formed by municipalities with a higher incidence of SG located in the North and Center-West regions in the period 2011–2015, and from the years 2016 to 2020 there was a modification of these high-high clusters in the study regions (Fig. 1).

### Spatial distribution of GS in the North region

**Fig. 2:**
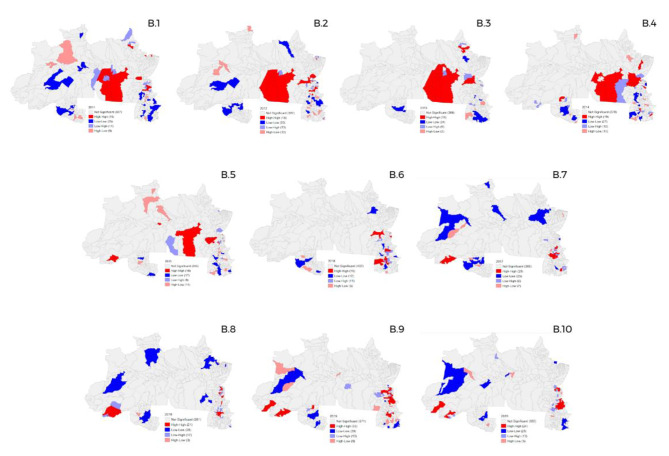
Spatial distribution of gestational syphilis cases in the North region of Brazil, from 2011 to 2020

In the North region of Brazil, the number of space conglomerates between 2011 and 2020 remained almost the same (Fig. 2), with a remarkable decrease in 2016 (B.6), but with a return on growth in later years. From 2011 to 2015 (Fig. 2 - B.1 to Fig. 2 - B.5), municipalities with greater territorial extension of this region had high-high and high-low clusters, a profile that changed in the following years, modifying to the smaller states of the North.

### Spatial distribution of GS in the Northeast region

**Fig. 3:**
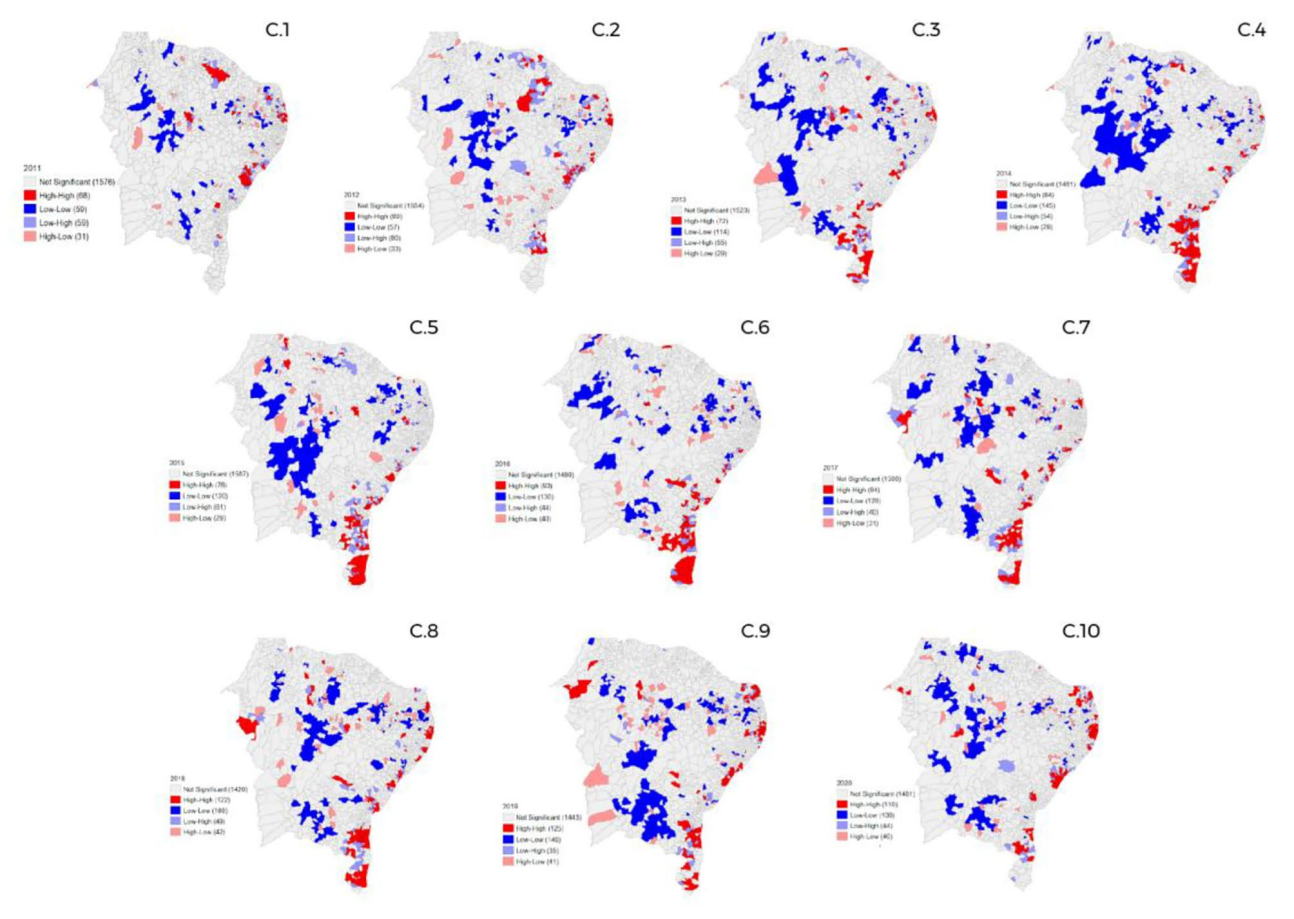
Spatial distribution of gestational syphilis cases in the Northeast region of Brazil from 2011 to 2020

Regarding the Northeast region (Fig. 3), the year 2018 (Fig. 3 - c.8) stands out as the year of the largest number of spatial conglomerates, mainly with regard to clusters considered high-high and low-low, a number that has been gradually reduced in the following years of the study.

### Spatial distribution of GS in the Center-West region

**Fig. 4:**
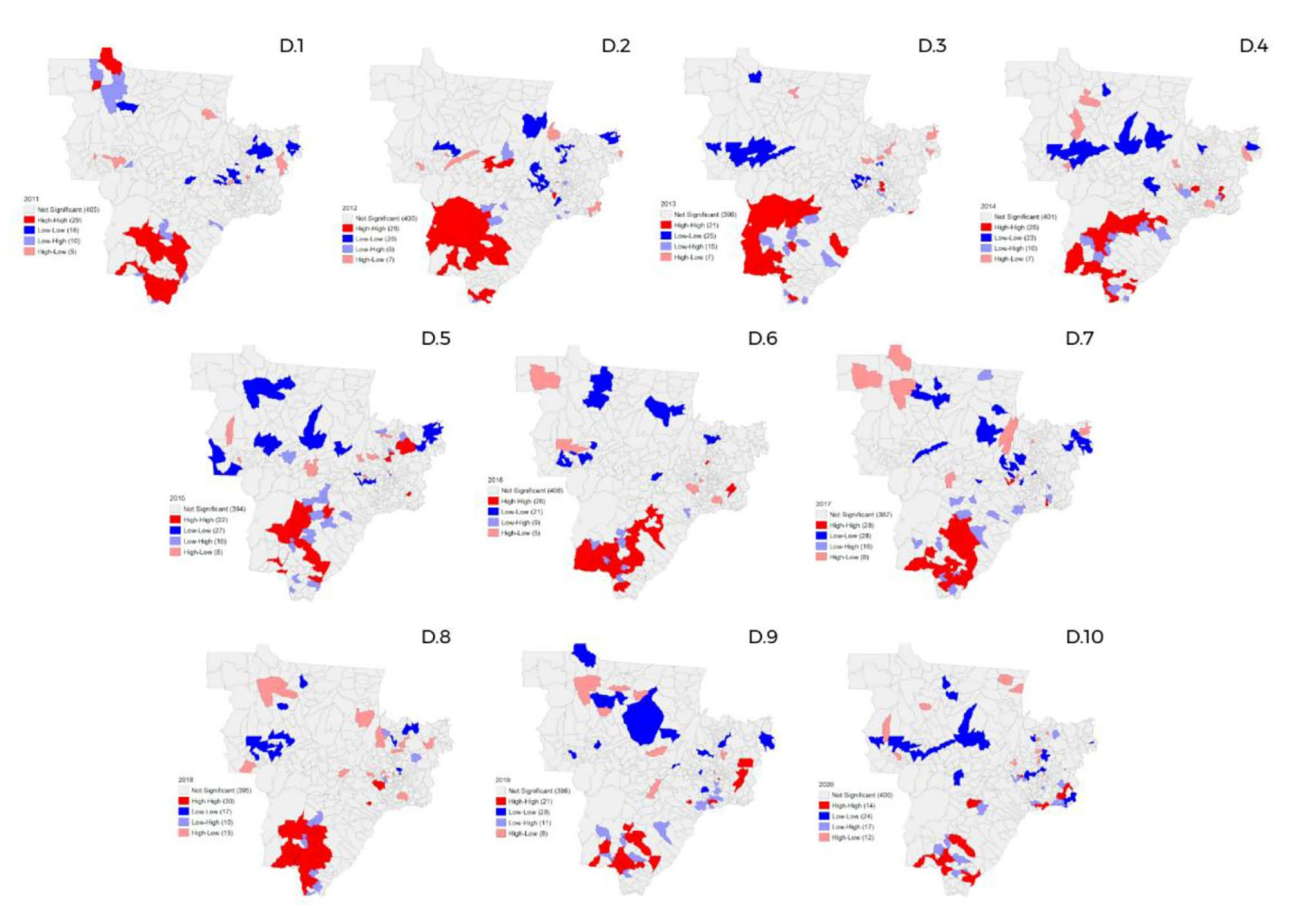
Spatial distribution of gestational syphilis cases in the Center-West region of Brazil, from 2011 to 2020

As to the Center-West Region (Fig. 4), it presented nearly the same number of space conglomerates in relation to the years 2011 and 2020, with small variations during that period. In 2020, the region had a significant reduction in high-high clusters compared to previous years (Fig. 4 - D.10). On the other hand, there was an increase in the high-low and low-high space conglomerates.

### Spatial distribution of GS in the Southeast region

**Fig. 5:**
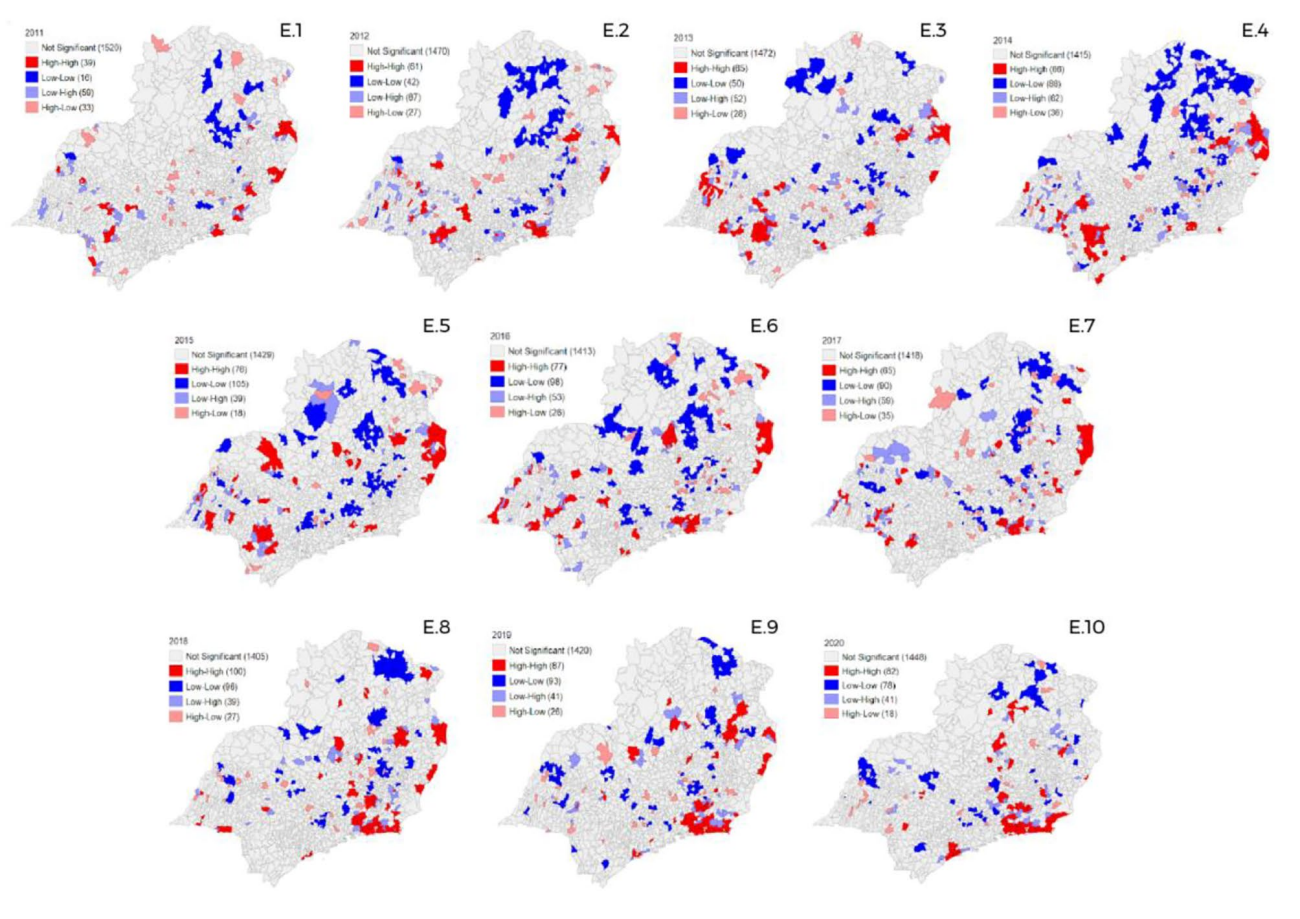
Spatial distribution of gestational syphilis cases in the Southeast region of Brazil, from 2011 to 2020

In Southeast Brazil, the number of space conglomerates was increased (Fig. 5), with 2011 and 2018 as the years of lower and higher number, respectively (Fig. 5 - E.1; Fig. 5 - E.7). It was verified the alternation of states, especially the state of Rio de Janeiro, which has had a growing high-high clusters since the year 2016. In contrast, Minas Gerais and Sao Paulo had more conglomerates in the years 2014 and 2015 (Fig. 5 - E.4; Fig. 5 - E.5).

### Spatial distribution of SG in the South region

**Fig. 6:**
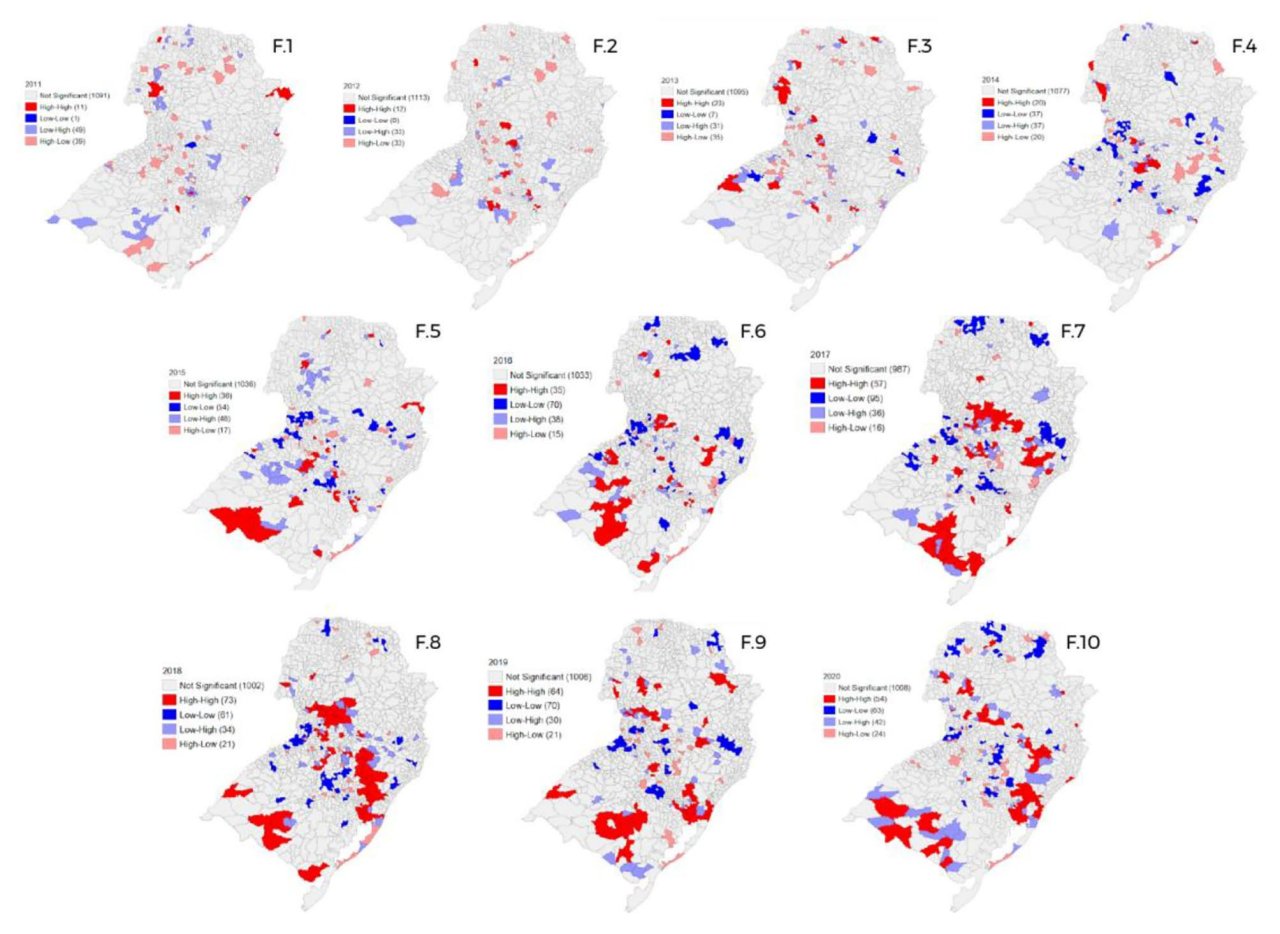
Spatial distribution of gestational syphilis cases in the South region of Brazil from 2011 to 2020

Finally, the South region of the country had the growth of the amount of space conglomerates in relation to the years 2011 and 2020 (Fig. 6 - F.1; F.10), with emphasis from the year 2015 on. In 2017 (Fig. 6 - F.7), this region presented more than half of the municipalities with most of them in high-high cluster conditions, a scenario presented mainly in the states of Santa Catarina and Rio Grande do Sul.

### Trend analysis of GS in Brazil

Brazil showed an increasing trend in gestational syphilis, with an APC of 19.07% (95%CI 13.92–24.46), while among the 5 regions, an increasing trend was observed in all of them, with a higher APC in: 22.55% of APC in the South, 19.71% of APC in the Northeast, 19.16% of APC in the Southeast, 17.95% of APC in the North and 17.89% of APC in the Centre-West (Table 1).

**Table 1:** Trend of cases of gestational syphilis notifications in Brazil from 2011 to 2020

Variable					
GESTATIONAL SYPHILIS	*p*-value	% annual percentage change	IC (95%) MIN	IC (95%) MAX	Trend
Brazil	**< 0.001**	19.07	13.92	24.46	**Increasing**
North	**< 0.001**	17.95	12.99	33.18	**Increasing**
Acre	**0.001**	24.63	13.56	36.77	**Increasing**
Amapá	**< 0.001**	22.1	16.39	28.1	**Increasing**
Amazonas	**0.001**	26.05	14.26	39.06	**Increasing**
Pará	**< 0.001**	13.54	11.92	17.71	**Increasing**
Rondônia	**< 0.001**	22.43	20.24	36.67	**Increasing**
Roraima	**< 0.001**	21.94	14.19	35.09	**Increasing**
Tocantins	**< 0.001**	23.99	15.44	21.96	**Increasing**
Northeast	**< 0.001**	19.71	15.17	35.73	**Increasing**
Alagoas	**< 0.001**	21.09	14.19	28.41	**Increasing**
Bahia	**0.016**	14.5	3.48	26.7	**Increasing**
Ceará	**< 0.001**	17.25	10.83	24.04	**Increasing**
Maranhão	**< 0.001**	25.02	15.6	26.55	**Increasing**
Paraíba	**0.009**	11.01	3.6	37.55	**Increasing**
Pernambuco	**< 0.001**	25.86	16.97	18.96	**Increasing**
Piauí	**< 0.001**	25.49	19.31	29.6	**Increasing**
Rio Grande do Norte	**0.002**	23.14	10.95	32	**Increasing**
Sergipe	**0.007**	13.21	4.83	38.08	**Increasing**
Center-West	**< 0.001**	17.89	15.08	20.76	**Increasing**
Goiás	**< 0.001**	20.01	15.91	24.04	**Increasing**
Mato Grosso	**< 0.001**	15.81	10.56	24.25	**Increasing**
Mato Grosso do Sul	**0.002**	11.64	5.89	35.2	**Increasing**
Federal District	**< 0.001**	31.6	27.6	22.27	**Increasing**
Southeast	**< 0.001**	19.16	14.29	24.24	**Increasing**
Espírito Santo	**0.15**	10.22	-4	26.7	Stationary
Minas Gerais	**< 0.001**	26.62	16.57	21.31	**Increasing**
São Paulo	**< 0.001**	16.7	11.68	30.21	**Increasing**
Rio de Janeiro	**< 0.001**	21.87	18.97	35.42	**Increasing**
South	**0.001**	22.55	12.47	23.13	**Increasing**
Paraná	**0.001**	20.04	11.18	15.19	**Increasing**
Rio Grande do Sul	**< 0.001**	24.34	14.44	24.84	**Increasing**
Santa Catarina	**0.002**	24.1	11.53	24.66	**Increasing**

Source: Developed by the authors based on the Brazilian Unified Health System - DATASUS database. Note: IC95% = 95% Confidence Interval

In relation to the states, 25 of the 27 Brazilian states showed an increasing trend. The greatest percentage variation was observed in the Federal District (31.6% PCA), followed by the state of Minas Gerais (26.62% PCA) and the state of Amazonas (26.05% PCA). It should be noted that only the state of Espírito Santo showed a stationary trend in the years analysed (Table 1).

## Discussion

The results indicate a growing trend of cases of GS in almost all of Brazil, as well as the identification of high-high clusters in different regions of the country. The disease had a considerable increase in cases during the period studied, a total of 279,618 cases of GS were reported in Brazil, with a peak of 37,436 cases in 2018 [16]. The national average incidence rate increased from 2.9 cases per 1,000 live births in 2011 to 5.5 cases per 1,000 live births in 2020 [16].

Data released in the United States also shows a growing trend in syphilis cases in that country, with an increase of 32% between 2020 and 2021, reaching the highest number of notifications in the last 70 years. Likewise, SG and SC cases advanced markedly during this period [21]. In Australia, the government implements public awareness campaigns about the prevention and treatment of syphilis infections, as, in 2022, notifications doubled in relation to the rates recorded in 2015 [22].

It is possible to affirm the resurgence of this STI throughout the world, as highlighted by the largest and most comprehensive study of syphilis genomes, including samples from 23 countries, from regions such as Western Europe, Central Asia and Africa [23].

According to data from this research, the spatial distribution shows a heterogeneous profile of notifications of the disease, with differences between regions, states and also intra-municipalities. This fact may be related to several factors, such as the availability of health services, the socioeconomic conditions of the population and access to information on disease prevention and treatment [24].

In the state of Espírito Santo, SG rates remained stationary. However, a study that investigated the spatial analysis of syphilis in pregnant women and congenital syphilis in the state of Espírito Santo, from 2011 to 2018, identified that this state also presents challenges in meeting the goal established by the WHO to control this infection, as they were identified areas with high incidence rates of congenital syphilis, gaps in prenatal care and underreporting of syphilis cases in pregnant women [25].

The increased incidence of GS in Brazil is a public health problem that deserves attention from health authorities, and late diagnosis of the disease and lack of treatment or inadequate treatment lead to vertical transmission and increased incidence of gestational syphilis [26]. The persistence of the disease is related to the lack of effective actions, since access to treatment and diagnosis are made available by the Unified Health System (SUS), and is therefore considered an avoidable problem [27].

It is expected that national strategies to combat syphilis such as the Stork Network and the Family Health Strategy (ESF), by contributing to the improvement of prenatal care (PN) and screening of women and their partners, will interfere with the increase in number of records of cases of the disease [27, 28]. However, this reality contradicts itself, since the existence of these strategies should also lead to an improvement in the resolution of diagnosed cases [27].

For this, one must reflect on the territorial extension of the country, which has a continental understanding, and this high population size highlights sociocultural differences and barriers in the efficient applicability of prenatal care, mainly in regions of difficult access or with low resources for care. to the needs of the population [11, 29].

It is also important to consider the context of the shortage of penicillin and other antibiotics used in the treatment of syphilis, which occurred between 2014 and 2017 in Brazil [26]. The lack of medication stock in the SUS services was detailed as one of the main causes of the resurgence of the disease, according to the Pan American Health Organization (PAHO) in 2016 [20]. In addition, the Covid-19 pandemic contributed to the decrease in adherence to PN consultations, and consequently to the screening and control of the disease [30].

In this context, studies suggest that the low incidence of STIs observed during the COVID-19 pandemic may be due to underdiagnosis and underreporting [31, 32]. But some countries, such as the United States and Canada, have reported a resurgence of syphilis, including congenital syphilis, following the COVID-19 pandemic [33, 34]. A retrospective descriptive study compared the number of syphilis cases before and after the COVID-19 pandemic in some countries in the Western Pacific Region of the World Health Organization. In 2021, China, Australia and Japan showed an increase in the number of cases of syphilis. This study warns of the possibility of an increase in cases of syphilis in the future around the world, requiring investment in prevention actions and control of STIs, such as syphilis, after the pandemic [35].

In addition, the lack of training of health professionals to identify and treat GS can lead to late or inadequate diagnoses [24]. Therefore, it is necessary to invest in the continuous training of health workers, to guarantee the skills and abilities necessary for qualified prenatal care, a fact this already recognized by some countries in Latin America, such as Argentina and Colombia [27].

Health education actions should also be inserted, to combat taboos and raise awareness of the need for diagnosis and control of the disease. For this, it is necessary to overcome barriers, such as the poverty present in several parts of the Brazilian territory, which makes it difficult to understand the health-disease process, intensifies the low adherence to the proposed treatment and the adoption of preventive practices [27].

A study carried out in 2018 points to a correlation between data from this study and the quality of Primary Health Care (ABS) in Brazil, both in terms of coverage and in relation to the dimensions of care [36]. As with notifications of GS, the states of Piauí and Rio de Janeiro had the best and worst rates of ABS quality, respectively [36–39].

This study has limitations inherent to the origin of the data, since it is based on passive notification of syphilis cases. The study’s limitation in the use of secondary data is recognized, which, given the possibility of underreporting, may underestimate the real epidemiological situation of syphilis. It should be noted that the data is updated periodically, which may alter the results depending on the dates of access. However, it is important to mention that, over time, the notification criteria have changed, and this has a direct impact on the variation in the number of notified cases. The possibility of underreporting the occurrence of gestational syphilis stands out. Finally, the value of secondary data that are available on public platforms is highlighted, which are often produced but underused, and can provide important information for understanding the national health scenario.

## Conclusion

The results found in this study are in line with data from the literature, which point to syphilis as an important public health problem, especially in regions such as Northern Brazil. The heterogeneous spatial distribution of the disease may be related to socioeconomic factors, such as low educational background, poverty, and lack of access to health services.

Gestational syphilis is a serious public health problem in Brazil. The numbers are alarming and show the urgent need for measures to prevent and control syphilis during pregnancy. One of the main challenges in combating congenital syphilis is the lack of early diagnosis and adequate treatment. Many pregnant women do not perform PN or are not tested for syphilis, which makes it difficult to identify the disease and prevent transmission to the fetus.

New studies of national scope are suggested on the subject of the study, the training of professionals to ensure the quality of prenatal care and the quality of syphilis notification records, and health education activities, especially in the most fragile regions regarding the disease.

## Data Availability

The data is public and available on the website <https://indicadoressifilis.aids.gov.br/>.

## References

[1] Paho. Organização Pan-Americana da Saúde - Organização Mundial da Saúde publica novas estimativas sobre sífilis congênita. https://www.paho.org/pt/noticias/28-2-2019-organizacao-mundial-da-saude-publica-novas-estimativas-sobre-sifilis-congenita#:~:text=28 de fevereiro de 2019

[2] Souza BSO et al. Análise epidemiológica de casos notificados de sífilis. Rev Soc Bras Clin Med. 2018 abr-jun;16(2):94 – 8.

[3] Lasagabaster MA, Guerra LO, Sífilis (2019). EIMC.

[4] Korenromp EL, Rowley J, Alonso M, Mello MB, Wijesooriya NS (2019). Correction: global burden of maternal and congenital syphilis and associated adverse birth outcomes—estimates for 2016 and progress since 2012. PLoS ONE.

[5] Bottura BR, Matuda L, Rodrigues PSS, do Amaral CMCA, Barbosa LG (2019). Perfil epidemiológico Da sífilis gestacional e congênita no brasil – período de 2007 a 2016. AMHFCMSCSP.

[6] Seabra I, Ferreira GRPN, Sorensen W, Oliveira C, Parente AT, Gir E, Reis RKK et al. Spatial scenery of congenital syphilis in Brazil between 2007 and 2018: an ecological study. 12:E058270. 10.1136/bmjopen-2021-058270. BMJ Open 2022. 10.1136/bmjopen-2021-058270 PMC9021778 35443962

[7] Amorim EKR, Matozinhos FP, Araujo LAS, Silva TPR. Tendência dos casos de sífilis gestacional e congênita em Minas Gerais, 2009–2019: um estudo ecológico. ESS. 2021;30(4). 10.1590/S1679-49742021000400006 34644777

[8] Kojima N, Klausner JD (2018). An update on the global epidemiology of syphilis. CER.

[9] Sully EA (2020). Adding it up: investing in sexual and Reproductive Health 2019.

[10] Sinan. Sistema de Informação de Agravos de Notificação. Ministério da Saúde. http://portalsinan.saude.gov.br/

[11] Nunes PS, Guimarães RA, Rosado LEP, Marinho TA, Aquino EC, Turchi MD. Tendência dos casos de sífilis gestacional e congênita em Goiás, 2007–2017: um estudo ecológico. ESS. 2021;30(1). 10.1590/S1679-49742021000100002 33503212

[12] Brasil (2021). Ministério Da Saúde. Secretaria De Vigilância em Saúde. Boletim Epidemiológico: Sífilis.

[13] Brasil. Ministério da Saúde. Secretaria de Vigilância em Saúde. Boletim Epidemiológico. Número Especial | Out. 2022.

[14] Barcelos MRB, Lima EFA, Dutra AF, Comerio T (2022). Avaliação do seguimento da sífilis congênita 2016–2019 em Vitória-ES. JHGD.

[15] Enny S, Paixao AndrêaJF, Ferreira JM, Pescarini, Kerry LM, Wong ML, Barreto LC, Rodrigues. Maria Yury Ichihara. Maternal and congenital syphilis attributable to ethnoracial inequalities: a national record-linkage longitudinal study of 15 million births in Brazil. Lancet Glob Health 2023; 11: e1734–42. 10.1016/S2214-109X(23)00405-9 10.1016/S2214-109X(23)00405-9 37858584

[16] Ramos R, de SP da S, Carneiro GR, de Oliveira ALS, da Cunha TN, Ramos VP. Incidence of congenital syphilis according to inequalities and living conditions in the city of Recife, Pernambuco, Brazil. Rev Bras Saude Mater Infant. 2021Jul;21(3):785–94. 10.1590/1806-93042021000300004

[17] IBGE – Instituto Brasileiro de Geografia e Estatística. Panorama Brasileiro. Rio de Janeiro: IBGE, 2021. https://cidades.ibge.gov.br/brasil/panorama

[18] Brasil. Ministério da Saúde. Banco de dados do Sistema Único De Saúde - DATASUS. http://www.datasus.gov.br

[19] Anselin L (1995). Local Indicators of Spatial Association. Geogr Anal.

[20] Antunes JLF, Cardoso MRA (2015). Uso Da análise de séries temporais em estudos epidemiológicos. ESS.

[21] Ramchandani MS, Cannon CA, Marra CM, Syphilis. A Modern Resurgence [published online ahead of print, 2023 Mar 31]. Infect. Dis. Clin. North Am., v. 37, n. 2, pp. 195–222, jun. 2023. Disponível em: https://pubmed.ncbi.nlm.nih.gov/37005164/10.1016/j.idc.2023.02.006 37005164

[22] Phua G, White C. The resurgence of syphilis in Australia. AJGP. Volume 53, Issue 3, March 2024. 10.31128/AJGP-08-23-6943 10.31128/AJGP-08-23-6943 38437656

[23] Beale MA, Marks M, Cole MJ (2021). Global phylogeny of Treponema pallidum lineages reveals recent expansion and spread of contemporary syphilis. Nat Microbiol.

[24] Araujo RS, Souza ASS, Braga JU (2020). A Quem Afetou o Desabastecimento De Penicilina para sífilis no Rio De Janeiro, 2013–2017?. RSP.

[25] Soares KKS, Prado TN, do., Zandonade E, Moreira-Silva SF, Miranda AE. Spatial analysis of syphilis in pregnancy and congenital syphilis in the state of Espírito Santo, Brazil, 2011–2018. Epidemiologia E Serviços De Saúde. 2020;29(1):e2018193. 10.5123/S1679-49742020000100018 10.5123/s1679-49742020000100018 32490938

[26] Jesus CM. Interface entre aspectos socioculturais e práticas de cuidado de profissionais de saúde em puérperas com sífilis: revisão integrativa de literatura. RI. 2019. https://app.uff.br/riuff/handle/1/22211

[27] Ribeiro RS, Segura GS, Mota ACF, Santos NSGM (2020). Epidemiologia Da sífilis gestacional e congênita: revisão integrativa de literatura. RSD.

[28] Miranda AE, Freitas FLS (2021). Políticas Públicas em infecções sexualmente transmissíveis no Brasil. ESS.

[29] Silva JL (2022). Distribuição Espacial Da sífilis gestacional na região Norte do Brasil de 2011 a 2020. RBE.

[30] Domingues RMSM (2015). Et Al. Adequação Da assistência pré-natal segundo as características maternas no Brasil. RPSP.

[31] Sentis A, Prats-Uribe A, López-Corbeto E (2021). O impacto da pandemia de COVID-19 nos dados de vigilância de Infecções sexualmente Transmissíveis: queda de incidência ou artefato?. BMC Saúde Pública.

[32] Berzkalns A, Thibault CS, Barbee LA, Golden MR, Khosropour C, Kerani RP (2021). Diminuição Nas infecções sexualmente transmissíveis relatadas durante o período de COVID-19 em King County, WA: Diminuição Da transmissão Ou Triagem?. Sexo Transm Dis.

[33] Nazir A, Masood W, Ahmad S (2022). Aumento do Aumento Da sífilis em meio à Pandemia De COVID-19 nos EUA: uma preocupação negligenciada. Ann Med Surg (Londres).

[34] The Public Health Agency of Canada. Infectious syphilis and congenital syphilis in Canada, 2021. December 2022. https://www.canada.ca/en/public-health/services/reports-publications/canada-communicable-disease-report-ccdr/monthly-issue/2022-48/issue-11-12-november-december-2022/infectious-congenital-syphilis-canada-2021.html

[35] Komori A, Mori H, Xie W, Valenti S, Naito T (2024). Rapid resurgence of syphilis in Japan after the COVID-19 pandemic: a descriptive study. PLoS ONE.

[36] Brasil (2019). Ministério Da Saúde. Secretaria De Vigilância em Saúde. Departamento De Vigilância, Prevenção E Controle das Infecções Sexualmente Transmissíveis, do HIV/Aids e das Hepatites Virais. Boletim Epidemiológico - Sífilis 2019.

[37] Sousa AC (2023). Et Al. Distribuição Espacial Da sífilis gestacional na Região Centro-Oeste do Brasil De 2011 a 2020. RBSMI.

[38] Soares KKS, Prado TN, Zandonade E, Moreira-Silva SF, Miranda AE. Análise Espacial Da sífilis em gestantes e sífilis congênita no estado do Espírito Santo, 2011–2018. ESS. 2020;29(1). 10.5123/s1679-49742020000100018 32490938

[39] Ruberite JA. Análise espacial e espaço temporal dos casos de sífilis em gestante e sífilis congênita nos municípios do estado de São Paulo, de 2010 a 2016. Tese. Faculdade de Saúde Pública. Universidade de São Paulo. 2020.

